# Intelligence, alcohol consumption, and adverse consequences. A study of young Norwegian men

**DOI:** 10.1177/1403494820944719

**Published:** 2020-09-11

**Authors:** Adrian F. Rogne, Willy Pedersen, Tilmann Von Soest

**Affiliations:** 1 Department of Sociology and Human Geography, University of Oslo, Norway; 2 Norwegian Social Research, OsloMet – Oslo Metropolitan University; 3 Department of Psychology, University of Oslo, Norway

**Keywords:** Intelligence, alcohol, intoxication, Norway, consequences, young adults

## Abstract

**Aims:**

Research suggests that intelligence is positively related to alcohol consumption. However, some studies of people born around 1950, particularly from Sweden, have reported that higher intelligence is associated with *lower* consumption and *fewer* alcohol-related problems. We investigated the relationships between intelligence, alcohol consumption, and adverse consequences of drinking in young men from Norway (a neighboring Scandinavian country) born in the late 1970s.

**Methods:**

This analysis was based on the population-based Young in Norway Longitudinal Study. Our sample included young men who had been followed from their mid-teens until their late 20s (*n* = 1126). Measures included self-reported alcohol consumption/intoxication, alcohol use disorders (AUDIT), and a scale measuring adverse consequences of drinking. Controls included family background, parental bonding, and parents’ and peers’ drinking. Intelligence test scores—scaled in 9 “stanines” (population mean of 5 and standard deviation of 2)—were taken from conscription assessment.

**Results:**

Men with higher intelligence scores reported average drinking frequency and slightly fewer adverse consequences in their early 20s. In their late 20s, they reported more frequent drinking than men with lower intelligence scores (0.30 more occasions per week, per stanine, age adjusted; 95% CI: 0.12 to 0. 49). Intelligence was not associated with intoxication frequency at any age and did not moderate the relationships between drinking frequency and adverse consequences.

**Conclusions:**

**Our results suggest that the relationship between intelligence and drinking frequency is age dependent. Discrepancies with earlier findings from Sweden may be driven by changes in drinking patterns.**

## Introduction

Intelligence predicts morbidity and mortality, even after controlling for socioeconomic variables [1,2]. In other words, people with higher intelligence tend to be healthier and live longer. It has been suggested that high intelligence enhances care of one’s own health and, thus, helps people prevent unhealthy lifestyles and chronic diseases [3], while low intelligence may increase the likelihood of poor habits that may undermine mental and physical health. Intelligence may also affect educational attainment, family relations, social and occupational status, and income [4,5], which may contribute to an intelligence gradient in the exposure to a wide range of health risks. However, alcohol seems to be an exception from the general observation that high intelligence may play a protective role for health. Although alcohol consumption is an established risk factor for a wide range of health problems, several studies have found a *positive* association between measures of intelligence and alcohol consumption [6-14]. We attempt to shed light on this somewhat puzzling relationship by studying two different age groups in a new national context, differentiating between alcohol consumption and adverse consequences of drinking.

### Intelligence, alcohol, and adverse consequences

As noted, several studies have found a positive association between intelligence and alcohol consumption. In two different UK cohort studies, high childhood intelligence predicted more frequent drinking [6] and lifetime problem drinking [6,7]. In another study, intelligence was positively associated with alcohol consumption in both the UK and the USA [9]. Moreover, high intelligence has been linked to earlier alcohol debut in the USA [10]. Other studies tend to find that moderate alcohol consumption predicts better cognitive abilities [11-13]. One possible explanation for this positive association is intelligence-related selection into educations and occupations where frequent drinking is more common. Whereas both genetics and other family background characteristics may confound the relationship between intelligence and alcohol consumption, a US twin study reported cognitive ability to predict alcohol consumption independently of family background and genetics [14].

Some studies have produced null, mixed, or contradicting results [[8, 15-18]. Among these, some used indicators of drinking habits that may rather be conceptualized as indicators of adverse consequences from drinking. Four Swedish studies that examined cohorts born in the 1950s reported negative relationships between high intelligence and various alcohol measures. One study of male conscripts showed that low intelligence predicted both high weekly intake of alcohol and binge drinking [19]. Another study found negative associations between intelligence and alcohol-related morbidity (among men) and mortality (among men and women) [20]. A third study reported a weak negative relationship between high intelligence and self-reported drunkenness in adolescence, and a stronger negative association with registered alcohol problems in adolescence and early adulthood [21]. A fourth Swedish study found a cluster indicating low intelligence to be associated with police records of hazardous drinking in young adulthood [18].

Thus, reported exceptions to the positive association between intelligence and alcohol use seem to fall into two partially overlapping categories. One comprises Swedish studies based on cohorts born around 1950 who entered their late teenage years and young adulthood in the late 1960s and early 1970s [18-21]. The other includes studies whose outcomes can be seen as indicators of adverse consequences of drinking [16,18,20,21].

### Drinking cultures

One reason why studies from Sweden have reported findings contrary to those from other parts of Europe and the USA may be that the association between intelligence and alcohol consumption is contingent on context and drinking culture. In the late 1960s and early 1970s, when these cohorts started to drink, both Sweden and Norway were “unintegrated” drinking cultures, with low population levels of drinking but high levels of binge drinking, alcohol-related harm, violence, and arrests for public drunkenness during weekends [22]. Swedish and Norwegian drinking culture was characterized by a high consumption of spirits, in contrast to the wine- and beer-dominated drinking cultures in central and southern Europe. The link between consumption and harm levels also differed between these cultures [23]. In the 1990s and early 2000s, there was a strong increase in alcohol consumption in Norway that was only partially accompanied by increases in harm rates [24]. This increase was driven largely by increased wine consumption, on weekdays and often linked to meals, which reflected a more “continental” drinking pattern on weekdays, and a convergence of drinking patterns across European countries.

### Drinking versus consequences

Considering why some studies have found negative associations between intelligence and adverse consequences of drinking, we speculate that the presumably protective role of intelligence may function differently with regard to alcohol than to other aspects of health behavior. Unlike many other health risk factors, many adverse consequences related to alcohol consumption are short term and relate to people’s behavior while under the influence (violence, accidents, etc.) rather than the long-term physiological or psychological effects of consumption. If intelligence generally is protective against health risks, intelligence may perhaps moderate people’s behavior while drinking, rather than moderating the amount and frequency of drinking itself. Higher intelligence may be associated with a greater degree of self-control over intake, behavior while under the influence, and/or exposure to risky situations. If so, drinkers with higher intelligence may more often avoid adverse consequences of their drinking. Such a pattern of positive correlations with consumption but negative correlations with alcohol-related harm has previously been observed for socioeconomic status, and the notion that high SES groups consume more alcohol but experience less alcohol-related problems has been denoted the “Alcohol Harm Paradox” [25].

### Aims

We investigate the association between intelligence and drinking patterns among young Norwegian men born in the 1970s. This is a relevant group due to their relatively high levels of alcohol-related problems, and because drinking patterns and lifestyles often consolidate in early adulthood [26]. A positive association between intelligence and drinking would be in accordance with the main findings in the literature, while a negative association would be consistent with findings from older Swedish cohorts. We also investigate whether men with higher intelligence experience fewer adverse consequences of drinking and whether intelligence moderates the associations between drinking frequently and experiencing adverse consequences.

## Data and methods

### Data

We use data from the Young in Norway (YiN) panel study [27]. Participants were recruited through schools in 1992 (T1), with follow-ups in 1994 (T2), 1999 (T3), and 2005–2006 (T4). Except for the control variables measured at T1, we analyzed data from T3 and T4 in this article. The response rate at T3 was 83.8% (*n* = 2923). Data collection for T3 was postal. Only participants who had completed the questionnaire in school at T2 (i.e. those who had not left school or changed schools between T1 and T2) were contacted to participate at T3. At T4, 2890 participants completed the questionnaire (response rate: 82.4%).

Survey data were linked to register data on parents’ educational level and intelligence test scores from the Norwegian Armed Forces’ conscript assessment (mandatory for males only). Some men in our sample did not complete this test, likely including those deemed unfit for service before the assessment took place. In our analyses, we included all male respondents with valid IDs and non-missing conscription test scores at T3 (*n* = 968) and T4 (*n* = 1126). Since intelligence and several other variables are measured only once, we cannot exploit the panel structure of our data and therefore conduct separate analyses for our outcome variables at T3 and T4. Written consent, and parental consent for those below age 15, was obtained from all participants at T1. The YiN study was approved by the Norwegian Data Inspectorate and the Regional Committee for Medical Research Ethics (approval no. S-05030).

### Measures

At both T3 and T4, we measured 4-week drinking frequency (“How many times during the last 4 weeks have you been drinking more than a few sips of alcohol?”), which was measured numerically. We top-coded all values above 28. We also measured 12-month frequency of intoxication by asking respondents to indicate how often they had drunk so much that they clearly felt drunk. The response options were “0 times,” “1 time,” “2–5 times,” “6–10 times,” “11–15 times,” and “more than 50 times.”

At T3 we used five items from the Rutgers Alcohol Problem Index (RAPI) [28], which is an instrument used to measure alcohol problems among young people, with slightly modified wordings. These five items are provided as Supplemental Material (S1). From these, we calculated the mean score (values ranging from 1 to 5, Cronbach’s α = 0.61). In the analyses, we standardized this scale, which we called the “adverse consequences scale,” to have a mean of 0 and a standard deviation (SD) of 1.

At T4, when participants were aged around 28 years, we used the 10-item Alcohol Use Disorders Identification Test (AUDIT) [29] to measure alcohol problems. The AUDIT is scored between 0 and 40, and higher values indicate a higher risk of hazardous or harmful alcohol consumption. Respondents with item non-response were treated as missing. To avoid drawing tautological conclusions, we omitted questions about alcohol consumption (questions 1–3) from the AUDIT scale in the Supplemental Analyses (S2) where drinking frequency was included as an independent variable.

Intelligence was measured by the General Ability scores from the Norwegian Armed Forces’ conscript assessment, which is a composite score calculated from the three timed tests in Arithmetic (30 items), Figures (36 items), and Word Similarities (54 items). The Arithmetic and Word Similarities tests are similar to the Arithmetic and Vocabulary Test in the Wechsler Adult Intelligence Scale (WAIS). The Figures test is comparable to the Raven Progressive Matrices. Scores from the three tests were combined and then transformed into stanine scores (standardized scores with values 1–9). The reliability has been reported to be high, and the correlation between the General Ability score and the WAIS IQ has been reported as .73 in a sample of 48 Norwegian conscripts [30]. Given that intelligence is a highly stable trait, scores obtained at the age of 18 years provide a good proxy for intelligence at higher ages.

### Control variables

Parents’ educational level comprised four categories, linked from administrative register data. These indicated the highest educational level among parents when the respondent was 16 years old, plus an indicator of missing data. We included each parent’s employment status (six categories) and occupational class (five categories, plus unemployment). We also included a dummy variable to indicate whether the respondent lived with both parents to capture having experienced parents’ early divorce or living with a single parent. The Parental Bonding Instrument (PBI) is a 10-item additive scale version of the original [31], measuring the quality of the parent-child relationship. Our indicator of how often the respondent had ever seen their parents drunk (five categories, ranging from “Never” to “A few times a week”) was meant to capture exposure to parents’ drinking. The Conduct Problems Scale is an 18-item additive scale combining items measuring antisocial behavior. We also included a dummy variable to indicate whether the respondent had a best friend who “Usually drinks alcohol as often as once a week” during adolescence as an indicator of drinking habits in the respondents’ peer network. Except for parents’ education, all control variables were self-reported at T1.

### Analyses

We ran separate ordinary least-squares (OLS) regression models for each outcome at T3 and T4, and introduced our control variables in a stepwise fashion. For each outcome and survey wave, we ran four models:

- Model 1 included intelligence and age.
- Model 2 added controls for family background indicators.
- Model 3 added controls for the PBI and parents’ drinking at T1.
- Model 4 added controls for conduct problems and friend’s drinking.

We treated intelligence score stanines (values 1–9) and frequency of intoxication (values 0 (“Never”) to 5 (“50 times or more”)) as continuous variables in OLS regressions. Ordered logistic regression, in which these were treated categorically, produced substantively similar results (not shown). To make results comparable across models, we restricted the sample in each analysis to respondents without missing values on any variable included Model 4. This restriction did not substantively affect our results.

## Results

Descriptive statistics (mean, SD) for the main variables are shown in Table I. Descriptive statistics for control variables are included as Supplemental Material (S2) along with a visualization of the distribution of the main variables (S3). Overall, the respondents drank slightly less frequently at T3 when the respondents were around 22 years old (mean: 4.05 times during the last four weeks) than at T4 when respondents were around 28 years old (mean: 4.73 times) but were intoxicated slightly more often during the last year at T3 than T4 (mean: 3.1 vs 2.9 on a scale from 0 (“no times”) to 5 (“more than 50 times”)).

**Table I.** Descriptive statistics for the main study variables (*n* = 968 at T3, *n* = 1126 at T4).

Variable	Mean	SD	*N* respondents (non-missing)
Intelligence score	5.83	1.65	1083
Age, intelligence test (conscription)	18.00	0.56	1115
Age, T3	21.90	1.84	967
Age, T4	28.45	1.99	1123
4-week drinking frequency, T3	4.05	4.26	926
4-week drinking frequency, T4	4.73	4.58	1095
12-month intox. freq. (treated as continuous), T3	3.10	1.47	954
12-month intox. freq. (treated as continuous), T4	2.90	1.47	1114
Adverse consequences scale, T3	1.38	0.50	944
AUDIT score, T4	8.51	4.75	1075
AUDIT score without consumption items, T4	2.77	3.26	1075

*Note*: AUDIT = Alcohol Use Disorders Identification Test.

To examine the relationship between intelligence and alcohol consumption, we regressed 4-week drinking frequency on intelligence scores while controlling for age (Model 1). The results are shown in Table II. We found a positive but small and non-significant association between intelligence score and drinking frequency around age 22 years (Panel 1). However, around age 28 (Panel 2), we observed a significant positive association between intelligence score and drinking frequency; a 1 stanine higher intelligence score corresponded to drinking on 0.30 more occasions over the past 4 weeks (age adjusted; 95% CI: 0.12 to 0.49). This association was reduced by a third after control for family background characteristics measured in adolescence (Model 2, Panel 2). After including other controls, the point estimates were no longer statistically significant but remained positive (Model 4, Panel 2).

**Table II.** OLS regression results for the associations between intelligence test scores and 4-week drinking frequency at T3 and T4.

	Model 1	Model 2	Model 3	Model 4
	Coefficient	Coefficient	Coefficient	Coefficient
	(robust SE)	(robust SE)	(robust SE)	(robust SE)
*Panel 1.*				
*Dependent variable: 4-week drinking frequency* (T3)				
Intelligence score (stanines)	0.067	0.002	0.025	0.023
	(0.085)	(0.093)	(0.092)	(0.092)
Observations	760	760	760	760
R^2^	0.002	0.048	0.077	0.082
Adjusted R^2^	−0.001	0.010	0.034	0.036
*Panel 2.*				
*Dependent variable: 4-week drinking frequency (T4)*				
Intelligence score (stanines)	0.304**	0.201*	0.194	0.192
	(0.092)	(0.100)	(0.101)	(0.100)
Observations	898	898	898	898
R^2^	0.012	0.045	0.057	0.060
Adjusted R^2^	0.010	0.012	0.019	0.019
*Controls:*				
Age	yes	yes	yes	yes
Parents’ educational level (register)		yes	yes	yes
Parents’ employment status at T1		yes	yes	yes
Parents’ occupational class at T1		yes	yes	yes
Lived with both parents at T1		yes	yes	yes
Parental Bonding Instrument (PBI)			yes	yes
How often seen parents drunk			yes	yes
Conduct problems scale				yes
Best friend drinks weekly				yes

Note: T1 is survey wave 1, when respondents were around 15 years old. T3 is survey wave 3, when respondents were around 22 years old. T4 is survey wave 4, when respondents were around 28 years old.

*p < 0.05; **p < 0.01.

We then regressed 12-month intoxication frequency on intelligence scores, separately by survey wave (Table III). We found no substantively or statistically significant associations in any model specification in either survey wave. This finding suggests that intelligence was not strongly related to heavy episodic drinking.

**Table III.** OLS regression results for the associations between intelligence test scores and 12-month intoxication frequency (measured categorically, treated as continuous) at T3 and T4.

	Model 1	Model 2	Model 3	Model 4
	Coefficient	Coefficient	Coefficient	Coefficient
	(robust SE)	(robust SE)	(robust SE)	(robust SE)
*Panel 1*				
*Dependent variable: 12-month intoxication frequency* (T3)				
Intelligence score (stanines)	0.005	0.005	0.014	0.010
	(0.035)	(0.036)	(0.036)	(0.035)
Observations	787	787	787	787
R^2^	0.014	0.066	0.102	0.146
Adjusted R^2^	0.012	0.029	0.060	0.104
*Panel 2*				
*Dependent variable: 12-month intoxication frequency (T4)*				
Intelligence score (stanines)	0.051	0.027	0.029	0.027
	(0.031)	(0.033)	(0.033)	(0.033)
Observations	915	915	915	915
R^2^	0.013	0.074	0.091	0.102
Adjusted R^2^	0.011	0.043	0.055	0.064
*Controls:*				
Age	yes	yes	yes	yes
Parents’ educational level (register)		yes	yes	yes
Parents’ employment status at T1		yes	yes	yes
Parents’ occupational class at T1		yes	yes	yes
Lived with both parents at T1		yes	yes	yes
Parental Bonding Instrument (PBI)			yes	yes
How often seen parents drunk			yes	yes
Conduct problems scale				yes
Best friend drinks weekly				yes

*Note*: T1 is survey wave 1, when respondents were around 15 years old. T3 is survey wave 3, when respondents were around 22 years old. T4 is survey wave 4, when respondents were around 28 years old.

No coefficients were statistically significant (*p* > 0.05).

Regressing the adverse consequences scale on intelligence revealed a weak, negative association when respondents were around age 22 (Table IV, Panel 1), where 1 stanine higher intelligence score was associated with a 0.05 point lower score on the standardized adverse consequences scale (age adjusted; 95% CI: –0.09 to –0.00). However, this association was statistically significant only in Model 1. No substantive association was found between intelligence scores and the AUDIT score at T4, when respondents were in their late 20s (Table IV, Panel 2). These findings suggest that intelligence is not an important predictor of hazardous or harmful alcohol consumption.

**Table IV.** OLS regression results for the association between intelligence test scores and standardized adverse consequences scale (T3) and AUDIT (T4).

	Model 1	Model 2	Model 3	Model 4
	Coefficient	Coefficient	Coefficient	Coefficient
	(robust SE)	(robust SE)	(robust SE)	(robust SE)
Panel 1				
*Dependent variable: Adverse consequences scale, standardized* (T3)				
Intelligence score (stanines)	−0.046*	−0.044	−0.037	−0.039
	(0.023)	(0.024)	(0.024)	(0.023)
Observations	777	777	777	777
R^2^	0.038	0.095	0.127	0.158
Adjusted R^2^	0.035	0.059	0.086	0.116
Panel 2				
*Dependent variable: AUDIT* (T4)				
Intelligence score (stanines)	−0.041	−0.056	−0.033	−0.045
	(0.099)	(0.106)	(0.106)	(0.104)
Observations	885	885	885	885
R^2^	0.006	0.039	0.075	0.094
Adjusted R^2^	0.003	0.005	0.037	0.054
Controls:				
Age	yes	yes	yes	yes
Parents’ educational level (register)		yes	yes	yes
Parents’ employment status at T1		yes	yes	yes
Parents’ occupational class at T1		yes	yes	yes
Lived with both parents at T1		yes	yes	yes
Parental Bonding Instrument (PBI)			yes	yes
How often seen parents drunk			yes	yes
Conduct problems scale				yes
Best friend drinks weekly				yes

*Note*: T1 is survey wave 1, when respondents were around 15 years old. T3 is survey wave 3, when respondents were around 22 years old. T4 is survey wave 4, when respondents were around 28 years old.

* *p* < 0.05.

Finally, we investigated whether intelligence may moderate the relationship between frequent drinking and experiencing adverse consequences of drinking. In this analysis, we regressed the adverse consequences scale at T3 (aged around 22) and the AUDIT instrument (excluding items on drinking from AUDIT to avoid tautological conclusions) at T4 (aged around 28) on drinking frequency and intelligence score and their interaction. The results showed only small, non-significant interaction terms (*p* > .05). These results are provided as Supplemental Material (S4) and provide little support for the notion that intelligence moderates the relationship between frequent drinking and experiencing adverse consequences from drinking.

In all analyses, intelligence and age explained very little of the variance in our outcomes, as measured by R^2^.

## Discussion and conclusions

In our sample of Norwegian men born in the 1970s, there was no association between intelligence and frequency of alcohol use when participants were in their early 20s. In their late 20s, we observed a positive association. However, we found no significant link between intelligence and intoxication frequency. Our findings are not consistent with findings from neighboring Sweden, which were based on cohorts born in the 1950s. Rather, our findings resemble those from other recent studies showing a positive association between intelligence and alcohol use, but this association is age-dependent and not very strong. One possible explanation may be that alcohol use in Norway changed considerably in the 1990s and early 2000s, when the so-called weekend binge drinking culture was supplemented by more frequent alcohol consumption. If intelligence is more positively associated with alcohol consumption in cultures with more frequent but less intensive drinking, such changes may have led to an emerging positive (or less negative) association between intelligence and drinking frequency. However, to disentangle these relationships, more research into changes in drinking patterns over time in relation to intelligence is necessary.

The positive association between intelligence and drinking frequency in the late 20s, when most men have entered the labor market, is also consistent with the notion that selection into longer educational programs or high-status jobs may be relevant to this association. Our findings also indicated a small, negative association between intelligence and alcohol related problems at around 22 years of age. While one possible interpretation of this finding is that high intelligence may protect against adverse consequences from drinking, additional analyses (not shown) indicate that the negative association is driven solely by higher adverse consequences scores in the two lowest stanines. Finally, our results do not support the notion that intelligence moderates the relationship between drinking frequency and adverse consequences of drinking.

Our study has several limitations. We cannot rule out the importance of selective attrition, measurement error, and similar survey-related issues. If people with greater cognitive abilities are more reflexive of, and concerned with, potentially adverse consequences of their drinking, they may be more likely to report alcohol use and related problems accurately in surveys [6], which may result in systematic measurement error. The group of nondrinkers may be highly diverse, possibly including both former heavy drinkers and lifetime abstainers [15]. Several of the included control variables in models 3 and 4 may also be affected by intelligence, drinking habits, or adverse consequences, and controlling for these may have introduced overcontrol bias. Our results may also be affected by reverse causation, since heavy alcohol consumption in adolescence may adversely affect cognitive ability [32]. Moreover, our data did not enable us to study women.

In conclusion, studying young Norwegian men born in the 1970s, our findings suggest that the association between intelligence and alcohol consumption is only positive when they are in their late 20s, not when they are in their early 20s. In other words, the association appears to be age dependent. This finding also contrasts with Swedish findings from older cohorts, suggesting that the relationship may also be context-dependent. Our results also suggest that intelligence does not moderate the relationship between frequent drinking and adverse consequences.

## Supplemental Material

SJP944719_Supplemental_Material – Supplemental material for Intelligence, alcohol consumption, and adverse consequences. A study of young Norwegian men Click here for additional data file. Supplemental material, SJP944719_Supplemental_Material for Intelligence, alcohol consumption, and adverse consequences. A study of young Norwegian men by Adrian F. Rogne, Willy Pedersen and Tilmann Von Soest in Scandinavian Journal of Public Health
