# Supplementary material for: Intelligence, alcohol consumption, and adverse consequences. A study of young Norwegian men
Source: Scand J Public Health. 2020 Sep 11;49(4):411–8. doi: 10.1177/1403494820944719 (PMC8135245; doi:10.1177/1403494820944719)
Supplement: SJP944719_Supplemental_Material – Supplemental material for Intelligence, alcohol consumption, and adverse consequences. A study of young Norwegian men [file SJP944719_Supplemental_Material.pdf]

Supplementary material

Intelligence, alcohol consumption and adverse consequences.

A study of young Norwegian men

S1: Question wordings for the adverse consequences scale

The items used for the adverse consequence scale at T3 are selected questions from the Rutgers Alcohol Problem Index (RAPI) [36].

At T3, respondents were asked

> *"Remember the past 12 months. How often during this time have you experienced the following problems related to your use of alcohol? (If you haven't been drinking, skip the questions)*
>
> *[1] Gotten involved in a fight, behaved badly or hurt someone*
>
> *[2a] Gone drunk to school or work*
>
> *[3] Tried to control the drinking by limiting it to certain hours or certain places*
>
> *[4] Suddenly appeared somewhere without remembering how you got there*
>
> *[5] Been told by a friend or neighbor that you should stop or limit your drinking".*

with response options "Never", "1 time", "2–4 times", "5–10 times" and "11 times or more".

Respondents who had not been drinking in the past 12 months were assigned to "Never". Response options were coded with values from 1 to 5. The additive scale was created by taking the mean response value for all five items, and setting the scale value to missing if one or more individual scale items was missing. Cronbach's α of the whole 5-item scale was 0.609.

S2: Descriptive statistics for control variables

Variable	Mean	SD	N respondents (non-missing)
Parental bonding instrument, T1	2.01	0.42	1042
Conduct problems scale, T1	0.41	0.41	1034
	Percent		
**Parental education (register)**			
University/college, long	14.02		157
University/college, short	30.89		346
High school/upper secondary (general or vocational)	47.68		534
Junior high school/comprehensive school or less	7.23		81
Missing register information on both parents	0.18		2
**Father's employment status at T1**			
Full-time	82.79		895
Part-time	4.63		50
Unemployed	1.76		19
Staying home	1.57		17
On welfare	1.76		19
Attending school, continuing education, or similar	1.20		13
Non-response	6.29		68
**Mother's employment status at T1**			
Full-time	51.53		557
Part-time	28.58		309
Unemployed	1.67		18
Staying home	9.25		100
On welfare	2.50		27
Attending school, continuing education, or similar	3.42		37
Non-response	3.05		33
**Father's occupational class at T1**			
Other/Unknown	5.92		64
Higher administrative occupation	13.23		143
Various middle positions	38.85		420
Lower functionary and more	6.11		66
Primary industry	6.01		65
Worker	25.07		271
Not employed	4.81		52
**Mother's occupational class at T1**			
Higher administrative occupation	2.10		22
Various middle positions	33.65		353
Lower functionary and more	35.37		371
Primary industry	1.81		19
Worker	11.15		117
Not employed	15.92		167
**Lived with both parents at T1**			
Yes	78.50		836
No	21.50		229
**How often seen parents drunk at T1**			
Never	55.46		579
A few times	29.31		306
A few times a year	10.15		106
A few times a month	3.54		37
A few times a week	1.53		16
**Best friend drinks weekly at T1**			
Yes	12.17		137
No	87.83		989

S3: Distribution of the main variables

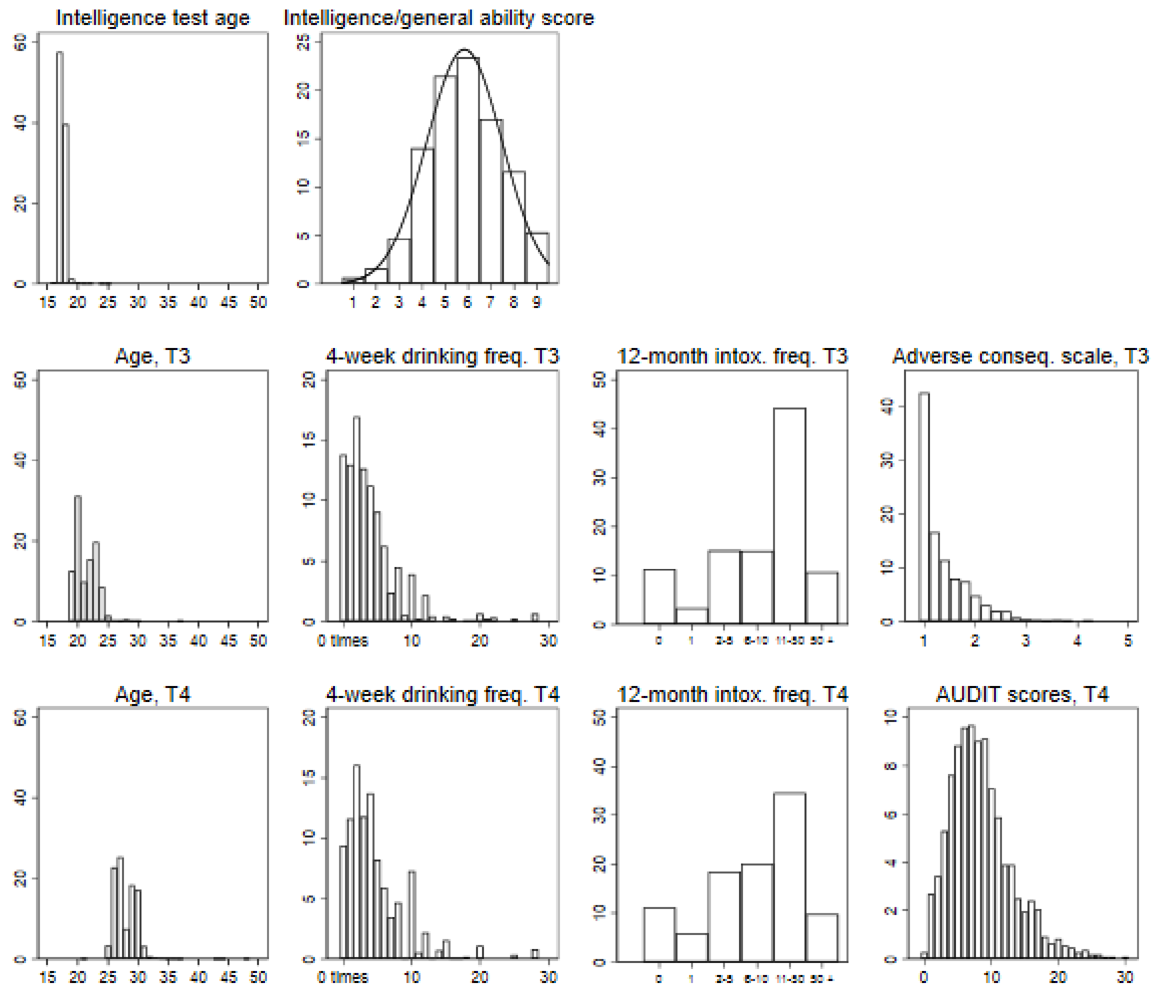

Note: All y-axes show percentages

S4: Supplementary analysis

OLS regression results for the associations between intelligence test scores interacted with 4-week drinking frequency, and standardized adverse consequences scale (T3) and AUDIT (T4).

	Model 1 Coefficient (robust SE)	Model 2 Coefficient (robust SE)	Model 3 Coefficient (robust SE)	Model 4 Coefficient (robust SE)
Panel 1				
Dependent variable: Adverse consequences scale, standardized				
Intelligence score (stanines)	-0.020	-0.009	-0.004	-0.006
	(0.030)	(0.031)	(0.031)	(0.031)
Drinking frequency (T3)	0.131*	0.133*	0.131*	0.127*
	(0.058)	(0.057)	(0.055)	(0.053)
Intelligence score (stanines) x Drinking frequency (T3)	-0.008	-0.009	-0.009	-0.009
	(0.009)	(0.008)	(0.008)	(0.008)
Observations	750	750	750	750
R^2	0.143	0.189	0.205	0.231
Adjusted R^2	0.139	0.154	0.165	0.190
Panel 2				
Dependent variable: AUDIT (without consumption items)				
Intelligence score (stanines)	0.022	0.027	0.071	0.073
	(0.084)	(0.090)	(0.090)	(0.090)
Drinking frequency (T4)	0.360**	0.357**	0.386***	0.393***
	(0.116)	(0.117)	(0.113)	(0.112)
Intelligence score (stanines) x Drinking frequency (T4)	-0.015	-0.015	-0.020	-0.021
	(0.019)	(0.019)	(0.019)	(0.019)
Observations	877	877	877	877
R^2	0.165	0.192	0.220	0.235
Adjusted R^2	0.161	0.161	0.185	0.199
Controls:				
Age	yes	yes	yes	yes
Parents' educational level (register)		yes	yes	yes
Parents' employment status at T1		yes	yes	yes
Parents' occupational class at T1		yes	yes	yes
Lived with both parents at T1		yes	yes	yes
Parental Bonding Instrument (PBI)			yes	yes
How often seen parents drunk			yes	yes
Conduct problems scale				yes
Best friend drinks weekly				yes

Note: T1 is survey wave 1, when respondents were around 15 years old. T3 is survey wave 3, when respondents were around 22 years old. T4 is survey wave 4, when respondents were around 28 years old.

*p < 0.05; **p < 0.01; ***p < 0.001
